# A novel device for cleaning the camera port during laparoscopic surgery

**DOI:** 10.1007/s00464-015-4229-3

**Published:** 2015-06-20

**Authors:** Eiji Kobayashi, Mamoru Kakuda, Yusuke Tanaka, Akiko Morimoto, Tomomi Egawa-Takata, Shinya Matsuzaki, Toshihiro Kimura, Yutaka Ueda, Kiyoshi Yoshino, Kiyokazu Nakajima, Tadashi Kimura

**Affiliations:** Department of Obstetrics and Gynecology, Osaka University Graduate School of Medicine, 2-2 Yamadaoka, Suita, Osaka, 565-0871 Japan; Department of Gastrointestinal Surgery, Osaka University Graduate School of Medicine, 2-2 Yamadaoka, Suita, Osaka, 565-0871 Japan

**Keywords:** Laparoscopic surgery, Camera port cleaner, Surgical device, Clear surgical image, Hindrance during surgery

## Abstract

**Aim:**

Poor visualization of the operative field due to an obscured camera lens is a problem frequently encountered while performing laparoscopic surgery. Little has been published about the prevention of lens obstruction specifically due to a contaminated camera port used for laparoscopic surgery. The aim of our study is to develop a new device, the Endowiper, for cleaning the laparoscopic port.

**Materials:**

The new cleaner for the port’s valve is made from rolled gauze. To simulate a surgical environment in the laboratory, we have used pseudo-blood to smudge the port’s valve.

**Method:**

In order to demonstrate the efficacy and safety of the Endowiper, we compared our method using this device with three previously reported port cleaning methods. These methods included use of gauze tightly wrapped around an endoscopic dissecting cramp, a small piece of gauze grasped by an endoscopic dissecting cramp, and a swab. We repeated the performance tests 280 times, 240 using a 12-mm trocar port and 40 with a 5-mm port.

**Results:**

With the 12-mm port, the complete port clearance rate achieved was 83.3 % by Endowiper, 56.7 % by wrapped gauze, 36.7 % by small gauze, and 40.0 % by swab. Trouble rate encountered during the procedure was 0 % by Endowiper, 1.7 % by wrapped gauze, 15 % by small gauze, and 90 % by swab. For the 5-mm port, the complete port clearance rate was 85 % by Endowiper and 20 % by sterile swab. The trouble rate was 0 % by Endowiper and 30 % by swab. Endowiper had a significantly superior result related to clearance rate than the other three methods in both the 12- (*p* < 0.001) and 5-mm (*p* < 0.001) ports. For trouble rate, Endowiper had a significantly superior result in both the 12- (*p* < 0.001) and 5-mm (*p* < 0.01) ports.

**Conclusion:**

This Endowiper will be an inexpensive device with a benefit to laparoscopic surgeons.

Condensation and debris on the camera lens due to a contaminated port are annoying problems encountered during laparoscopic surgery. Not only does lens contamination influence the surgical view, it also affects the surgeon’s mood. Methods for prevention of lens condensation have been well described in previous publications [1–3], whereas little has been written about methods to prevent lens contamination from the port [4]. We have developed a new camera port cleaner and compared the effectiveness of the device to that of previously reported instruments.

## Materials

**Fig. 1:**
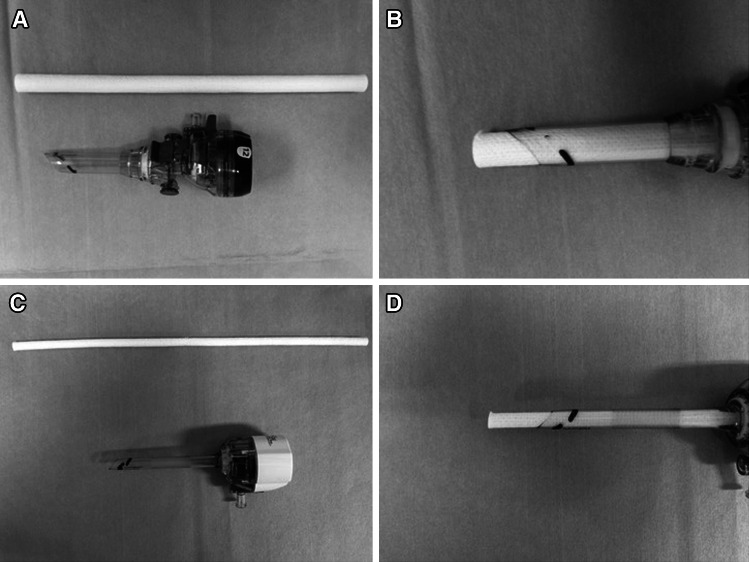
**A** 12 mm Endowiper, **B** the device introduced into a 12-mm port, **C** 5 mm Endowiper, **D** the device introduced into a 5-mm port

The camera port cleaning device, Endowiper (Osaki Medical Co., Ltd), is a tubular-type device made from tightly rolled cotton-like gauze. An X-ray-detectable thread is embedded in the core of the roll to assist in retrieval, in case of device loss in the abdomen. The length of the Endowiper is 25 cm; the diameter is either 5 or 12 mm (Fig. 1). The disposable device is intended for single use only. We used different kinds of ports from different manufactures: Johnson & Johnson B5LT 5 mm and B12LT 12 mm, Covidien NB5SHFLP 5 mm and 1179096P 12 mm, and the Applied Medical CTR22 5 mm and CTR73 12 mm ports. The structure of the valve in the B5LT, NB5SHFLP and 1179096P was 2-petaled, and in the B12LT, CTR22, and CTR73, it was 4-petaled.

### Pseudo-blood

**Fig. 2:**
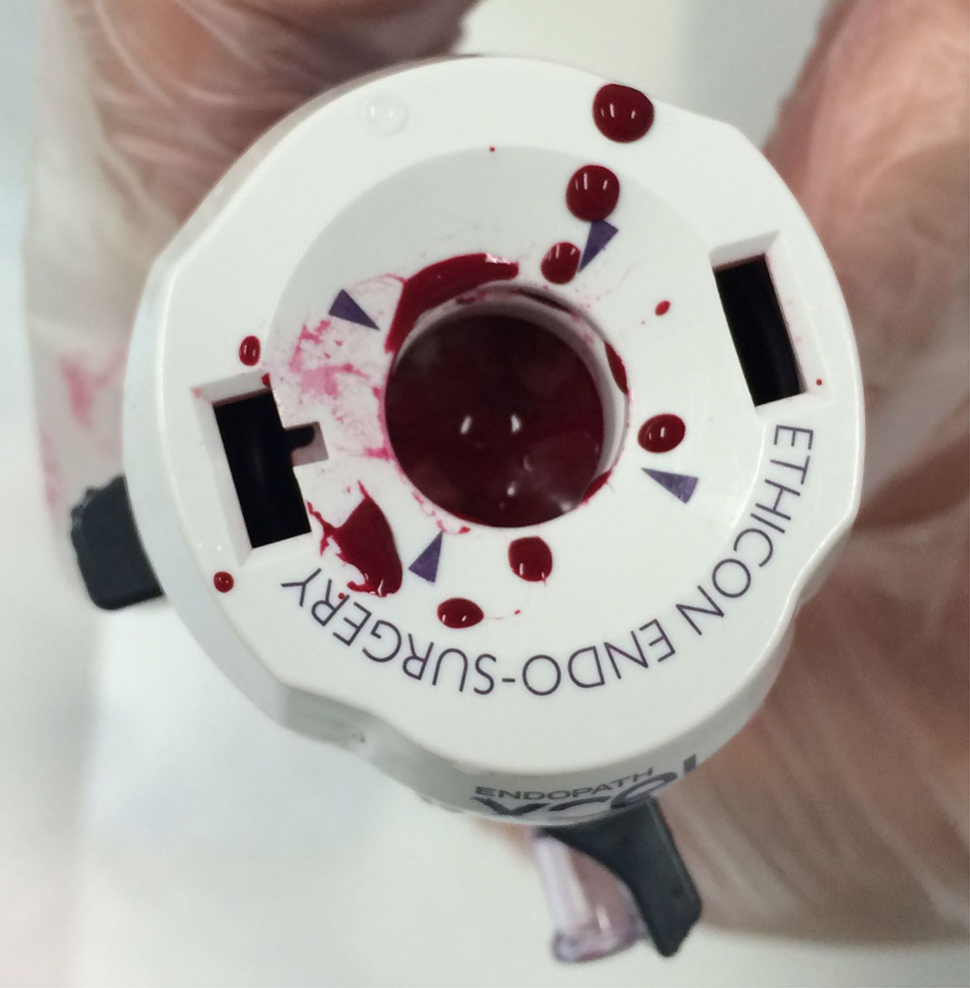
Contaminated port valve by pseudo-blood

To simulate the surgical environment in a laboratory, we used pseudo-blood to smudge the port’s valve (Fig. 2). Pseudo-blood cells (Yamashina Seiki Co., Ltd)are made from resin particles 8 μm in diameter, the same size as human red blood cells. The viscosity of the pseudo-blood plasma was adjusted to 2 millipascal second (mPa s), the same as blood plasma. Pseudo-blood cells and plasma were combined and adjusted to a hematocrit of 45 %, similar to human blood.

## Method

**Fig. 3:**
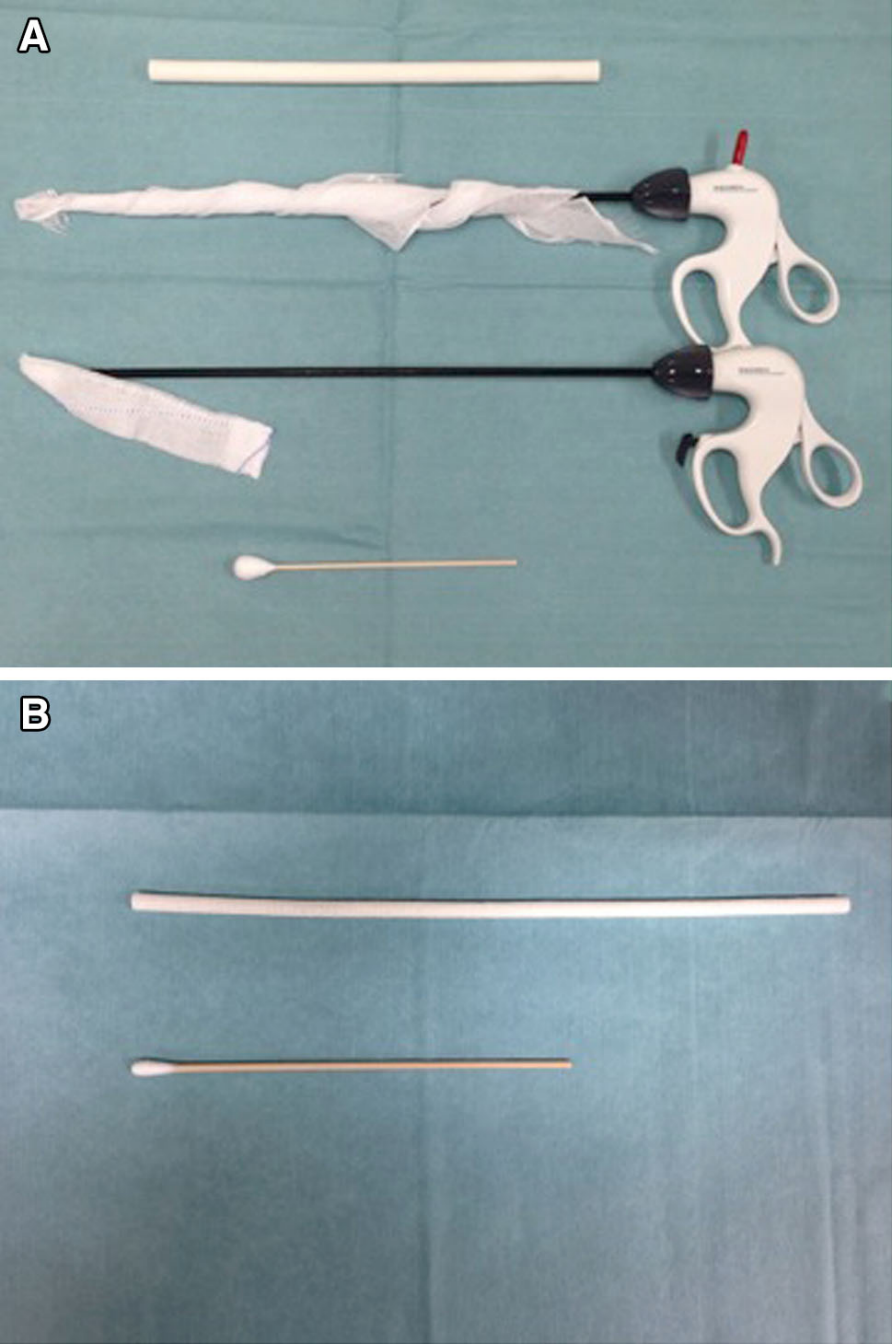
Port valve cleaning devices for 12 mm port (**A**) and 5 mm port (**B**). Image **A** shows an Endowiper, a sterile gauze tightly wrapped around an endoscopic dissecting cramp, a small sterile gauze grasped by an endoscopic dissecting cramp, and a sterile swab for a 12-mm port valve cleaner. Image **B** shows an Endowiper and a sterile swab for a 5-mm port valve cleaner

To demonstrate the efficacy of the Endowiper, we compared it with three previously reported port cleaning methods already in daily use (Fig. 3). The first method described is for the Endowiper, the second method uses a large sterile gauze tightly wrapped around an endoscopic dissecting cramp (large gauze), the third method is a small piece of sterile gauze grasped by an endoscopic dissecting cramp (small gauze), and the fourth method uses a 10- or 5-mm sterile cotton swab (swab).

To perform these tests under a controlled and reproducible condition, they were conducted in a surgical simulation in the laboratory, not in a human body.

We smudged both sides of the 5-mm port valve with 1 ml (total) of pseudo-blood; we used 2 ml for the 12-mm port. Half of the pseudo-blood was injected into the port inlet, the remainder into the outlet. During the procedure, the port was twice inverted in a 360° motion to uniformly smudge the valve. We inserted each cleaning device until it passed through and out of the port before removing it (Figure 1B, D).

**Fig. 4:**
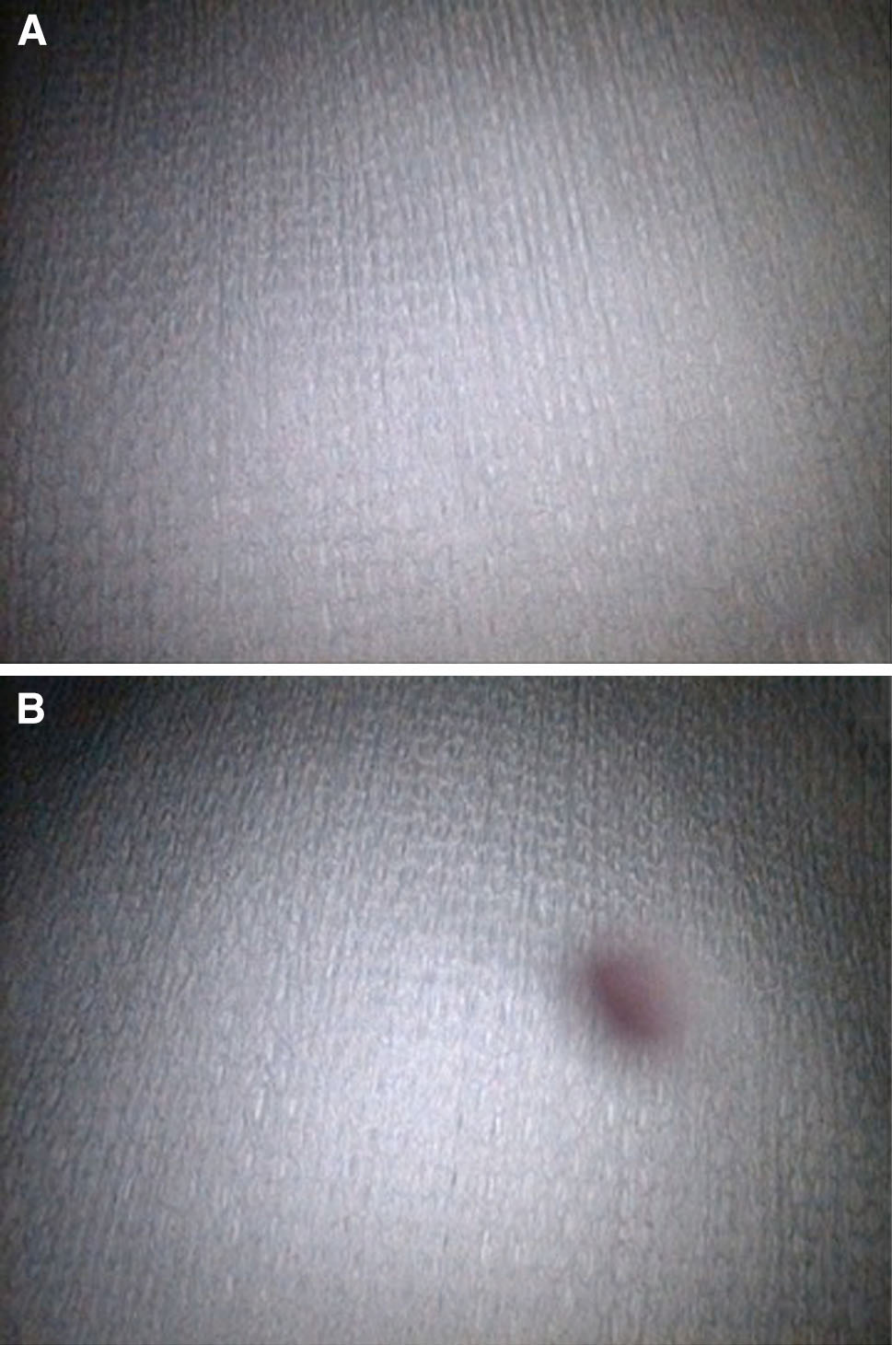
Camera images captured after cleaning the port valve. **A** Completely clear image; cleaning was judged effectively **B** blurry, obscured, or otherwise not completely clear image; cleaning was deemed ineffective

This procedure was performed twice for each cleaning device. Each cleaning device was used for each cleaning procedure. After cleaning of the port, we inserted a 0° laparoscope and captured the image to determine whether the cleaning method was effective or ineffective (Fig. 4). We defined the port cleaning results as either effective or ineffective based on the clarity of the laparoscopic image. A method was deemed effective if it resulted in a completely clear image. Methods resulting in images that were blurry or otherwise not completely clear were deemed ineffective. In addition, we enumerated the troubles encountered with each cleaning event. Trouble was defined as whether the material was caught at the valve or the material was torn during the test. To avoid bias from subjective judgement, the captured laparoscopic images were assessed in a blinded test by surgeons who are familiar with laparoscopic surgery. The captured images were shown in random order to two surgeons using a computerized system. To assess the effect of multiple uses, we performed durability tests during reusage.

### Statistical analysis

The Chi-square test was used to study associations between the type of device and its clearance or trouble rates. Secondly, we used logistic regression analysis to examine the association of the type of device and clarity of picture. Odds ratios (OR) and 95 % confidence intervals (CI) for clarity were calculated, and *p* values of <0.05 (two-sided test) were considered to be statistically significant. All analyses were performed using STATA 12.1 (StataCorp, College Station, TX, USA).

## Results

Table 1 shows the results of cleaning tests for both a 12- and 5-mm port valve. In total, we performed 280 tests, 240 times for the 12-mm trocar port and 40 times for the 5-mm trocar port. For the 12-mm port, the tests were conducted 60 times in each of the four devices. The complete port clearance rate was 83.3 % with the Endowiper, 56.7 % by large gauze, 36.7 % by small gauze, and 40 % by swab. Trouble rate during the procedure was 0 % by Endowiper, 1.7 % by large gauze, 15 % by small gauze, and 90 % by swab. The difference was statistically significant (*p* < 0.001) between the four groups.

**Table 1:** Clearance rate and trouble rate for four cleaning devices

Characteristics	5 mm trocar				12 mm trocar			
	N	(%)	Clearance rate (%)	Trouble rate (%)	N	(%)	Clearance rate (%)	Trouble rate (%)
All	40	100			240	100		
Type of device								
Endowiper	20	50.0	85.0	0.0	60	25.0	83.3	0.0
Gauze	N/A	N/A	N/A	N/A	60	25.0	56.7	1.7
Small gauze	N/A	N/A	N/A	N/A	60	25.0	36.7	15.0
Sterized swab	20	50.0	20.0	30.0	60	25.0	40.0	90.0
*p* value			*p* < 0.001	*p* < 0.01			*p* < 0.001	*p* < 0.001

For the 5-mm port, we conducted the tests for only two of the four devices, the Endowiper and the swab, because the large gauze and small gauze devices could not pass through the 5-mm trocar. Each test was done 20 times. The complete port clearance rate was 85 % for Endowiper and 20 % for the swab. The trouble rate was 0 % by Endowiper and 30 % by swab. The clearance and trouble rate were significantly different between the two groups (*p* < 0.001 and *p* < 0.01).

Table 2 shows associations between the type of devices and clarity achieved, separately by the port size. As for 5 mm port, we found that Endowiper was significantly associated with increased odds of clarity rate (OR 22.67; 95 % CI 4.34–117.47), compared to the sterilized swab. As for the 12-mm port, compared to gauze, Endowiper was significantly associated with clarity (OR 3.82; 95 % CI 1.63–8.94). In contrast to Endowiper, the swab was significantly associated with reduced odds of clarity (OR 0.44; 95 % CI 0.21–0.92). No clear associations were found for the small gauze.

**Table 2:** Odds ratios for clarity associated with type of device

Variables	5 mm trocar		12 mm trocar	
	OR	(95 % CI)	OR	(95 % CI)
Type of device				
Endowiper	22.67	(4.34–117.47)	3.82	(1.63–8.94)
Gauze	N/A		1.00	
Small gauze	N/A		0.51	(0.25–1.05)
Sterized swab	1.00		0.44	(0.21–0.92)

*CI* confidence interval, *NA* not applicable, *OR* odds ratio

We performed a durability test using a 5- or 12-mm Endowiper up to ten times to determine whether the images remained clear. Five different Endowiper devices were tested for each diameter. The median number of successful continuous uses of the Endowiper was twice for the 5-mm Endowiper and ten times for the 12-mm Endowiper, respectively.

## Discussion

During laparoscopic surgery, in the absence of any tactile input, the surgeon depends completely on the image transmitted by the laparoscope. Very minor contamination of the laparoscope lens leads to dramatic and progressive deterioration of the surgical image. Difficult visualization requires increasing mental effort to maintain a safe procedure, and the surgeon’s accuracy and speed progressively decrease [4]. Because we found little had been reported about efficient methods to prevent lens contamination, we developed a new camera port cleaner and compared the effectiveness between this instrument and previously reported instruments/methods.

Our results demonstrate that Endowiper is more effective at cleaning the port and has a lower trouble rate than three other popular methods. Endowiper has three strong points, compared with these other methods. The first is its greater port valve cleaning effectiveness. Because Endowiper is made from rolled gauze, the diameter is more uniform and consistent. We speculate that Endowiper is more uniformly cleaning both the inlet and outlet sides of the valve. In addition, the length of Endowiper is 25 cm; we speculate that it can thus clean the shaft from the inlet to the outlet of the port more uniformly than the small or large gauze devices.

Secondly, the Endowiper proved to be a safer valve cleaner than the other methods. The tubular shape of the device greatly reduces the risk of tearing loose gauze fragments that could present a problem. The other valve cleaning methods present a higher risk of leaving gauze fragments behind in the abdominal cavity, if the surgeon forcibly withdraws the cleaning device, which can cause tearing or catching of the gauze.

A risk of blindly inserting a cleaning gauze using a dissecting cramp is the possibility of incurring vital organ injury during the procedure. In contrast, the stiffness of the Endowiper is modest. Even if we insert Endowiper forcibly, the risk of vital organ injury is minimal. Lastly, the Endowiper has a relatively low cost (approximately $5). It is thus not a significant financial concern.

Although the development of the laparoscope was a remarkable achievement, development of peripheral equipment has not kept pace. This new device is the first device to be developed specifically for port cleaning. This device demonstrates superior efficacy and safety compared to previously reported methods.

A limitation of this study is that, for ethical reasons, it was not tested on a human body. Although our results may not reflect all possible surgical situations, the degree of smudging of the port’s valve depends on the skill of the laparoscopist and assistants. If the surgical team is skillful, the port will rarely be smudged. With less experienced surgical teams, the port will more often be smudged and cleaning of the port to gain a satisfactory surgical image may be required many times. Because our institute has many physicians and residents with different levels of training, we determined we could not perform the test in a consistent manner in routine practice. We decided to simulate the surgical environment in the laboratory using pseudo-blood to maintain consistency.

## Conclusion

This new device will be of great help, with little expense, for all of laparoscopic surgeons.
